# Carbohydrate-Based Micro/Nanocapsules With Controlled External Surface for Medical Applications

**DOI:** 10.3389/fchem.2020.00545

**Published:** 2020-06-26

**Authors:** Roman Bielski, Zbigniew J. Witczak, John F. L. Newport

**Affiliations:** ^1^Department of Pharmaceutical Sciences, Wilkes University, Wilkes Barre, PA, United States; ^2^Chemventive, LLC, Chadds Ford, PA, United States

**Keywords:** micro/nanocapsules, drug delivery, surface control, carbohydrate-derived monomers, interfacial polymerization

## Abstract

Micro/nanocapsules would have many more applications if we were able to controllably populate their surface with various chemical moieties. The present review introduces a novel variant of interfacial polymerization (IP) as a very robust method of manufacturing reservoir micro/nanocapsules equipped with several different functionalities on the capsules' surface. We call the method—IPCESCO (Interfacial Polymerization for Capsules' External Surface Control). As always in IP, the capsules' forming reaction is between monomers dissolved in opposite phases (oil or water) and takes place at the interface. Each monomer carries two or more functionalities reacting with functional groups of the monomer dissolved in the other phase. IPCESCO requires that one or both monomers are additionally equipped with (protected) functional groups interfering neither with the payload nor with the polymer formation. These additional groups end up everywhere in the polymeric shell but most importantly they are present on the external surface of capsules. These “handles” allow for the introduction of various moieties onto the capsules' surface. Since carbohydrate chemists developed a plurality of protecting and deprotecting methods for various functional groups such as aldehyde and hydroxyl, modified mono, and oligosaccharides are particularly well-suited to act as monomers in IPCESCO. The article discusses possible monomers and their synthesis, the transformation of protected reactive groups on the external capsules' surface into the desired functionalities, the control of the number of moieties on the surface and the capsules surface's architecture. The most important application of the novel encapsulation technology is in drug delivery. Possible surface units facilitating capsules' transport in the body, delivery to specific locations and mechanisms of capsules rupture are also addressed. Other applications of novel capsules include an ultra-sensitive quantitation and removal of pathogens, transport of nutrients in plants, detection of various antigens and other parameters in single cells.

## Introduction

Numerous applications of micro and nanocapsules are in the area of drug delivery, agriculture, textile and food industries. Most of the relevant research focuses on new ways of making capsules, novel components of the payload, and a better control of the rate of the payload release. Relatively little effort has been devoted to the introduction of various chemical moieties onto the external surface of capsules. However, it seems that capsules could find many new applications if we were able to equip their surface with several different functional groups or units. For example, if we could introduce moieties facilitating transport and “parking” of drug delivering capsules in the desired location we could minimize losses of drugs caused by the payload degradation in the body. Similarly, if we were able to introduce onto the capsules' surface antibodies recognizing all pathogenic antigens of concern in a given matrix, we possibly could measure and remove all these pathogenic cells or compounds from the system. Also, capsules with multifunctional surface should find various uses in ultra-sensitive detection.

The purpose of the present paper is to put forward a method allowing for an excellent control of various chemical moieties on the capsules' external surface. We call the method: **I**nterfacial **P**olymerization for **C**apsules **E**xternal **S**urface **Co**ntrol (IPCESCO). It is a modified interfacial condensation or addition polymerization.

## Interfacial Polymerization

Let us first look at the manufacture of micro(nano)capsules using traditional interfacial polymerization (Perignon et al., 2014; Piradashvili et al., 2016). In this process, a mixture of two immiscible liquids is used. Usually, the hydrophilic phase is composed of water (and some water-soluble compounds). The hydrophobic liquid (non-miscible with water) is often referred to as “oil.” The term–oil–may mean oil (triester of glycerol) but it also includes water-insoluble organic solvents. When a mixture of water and oil is vigorously mixed, an emulsion is formed. The size of droplets (of the dispersed phase) depends on the temperature, energy of stirring (or employed ultra-sound) and quantity and type of added surfactant(s). Thus, the size of droplets can be reasonably well-controlled. Depending on the relative quantities of oil and water we distinguish two types of emulsions: “oil in water” and “water in oil.” In a “water in oil” emulsion, oil is a continuous phase and, typically, the emulsion consists of substantially more oil than water. Obviously, in an “oil in water” emulsion, water is a continuous phase. The choice of the emulsion type depends mainly on the solubility of the future major component of the payload such as active pharmaceutical ingredient (API). It must end up in the dispersed phase (inside droplets). Incidentally, the polar solvent (not mixing with oil) does not have to be water (Crespy et al., 2007).

Now, we add two compounds to the emulsion: one, water-soluble, equipped with two or more X groups, and a second one, oil-soluble, equipped with two or more Y groups. If X reacts with Y the reaction will produce a polymer. Since both compounds are soluble in opposite phases the reaction must take place at the interface. Thus, the resulting solid polymer forms a shell surrounding the droplets. If the main compounds of interest such as active pharmaceutical ingredients (API) or reporter molecules (RM) were inside the droplets they become a payload (core) of formed capsules. Of course, any other compounds present in the dispersed phase will also become components of the payload.

The control of the shell thickness may be challenging since after some time reacting molecules (monomers) encounter increasing difficulties in reaching each other. However, a gradual temperature increase during the process and the use of adequate surfactants and phase transfer catalysts facilitates the contact between reacting molecules. The only property that is difficult to control is the capsules' size distribution, which is often not as good as when employing other encapsulation methods. However, an effective method of preparing uniform microparticles in relevant systems was described recently (Ma, 2014).

If the reaction between X and Y is an addition, the process is called interfacial addition polymerization (IAP). If the reaction between X and Y is a condensation, the process is called interfacial condensation polymerization (ICP) or interfacial polycondensation. While IP may not be the most convenient method of manufacturing nano/microcapsules, it offers something fundamental: the possibility of designing and controlling the outer surface of capsules.

## IPCESCO

**Figure 1:**
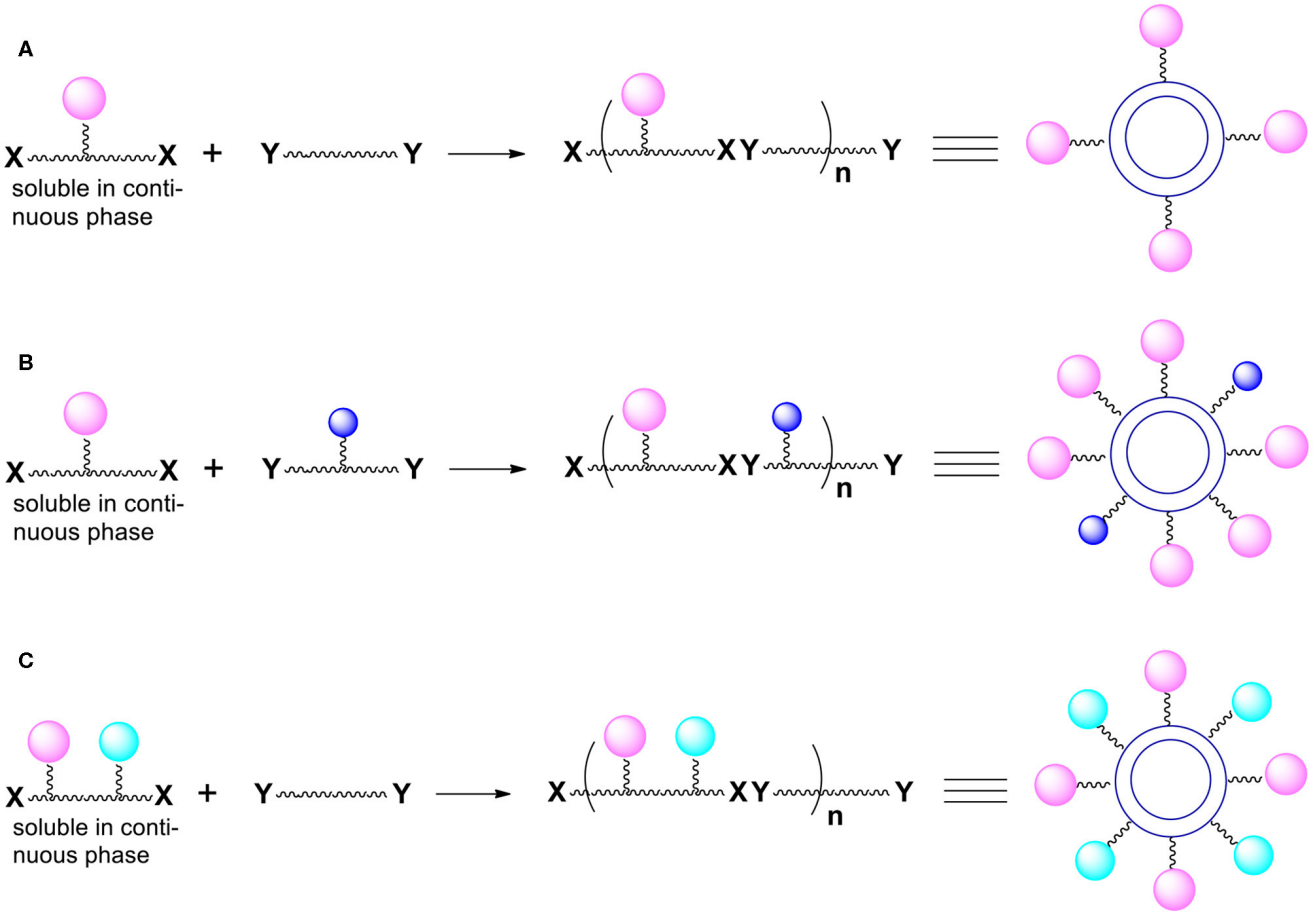
Introduction of functional groups onto the external surface of reservoir nano/microcapsules. **(A)** Additional groups (pink spheres) are present only in monomer molecules that are soluble in the continuous phase. **(B)** Additional groups (pink and blue spheres) derive from both bi(poly)functional monomers. **(C)** Additional groups (pink and green spheres) derive from one of the monomers (the one that is soluble in the continuous phase).

This control can be accomplished by introducing chemical functionalities we call “handles” onto the capsules surface (Bielski and Witczak, 2017). The presented here perspective expands the potential synthetic schemes and applications of the novel method of manufacturing micro/nanocapsules. The desired moieties we want to be present on the capsules surface are later attached to the “handles.” To be able to introduce “handles” onto the external surface of capsules, we must use monomers (during polymerization) equipped not only with reactive groups X and Y but also with other functional groups not involved in the polymerization process. Thus, imagine that one or both bifunctional compounds (monomers) contain, somewhere between two Xs or two Ys, additional reactive functionalities (pink, blue and green spheres; Figure 1) that are not capable of reacting with X and Y (for example, as a result of the use of protective groups).

The resulting polymer which forms the capsules' shell contains various functionalities represented by colored spheres on the Figure 1. The functionalities are everywhere within the polymer (on the internal surface of the shell, within the shell itself and on the outside surface of the shell). What is particularly important is that there are moieties (colored spheres) on the external surface of capsules. Note that the monomer dissolved in the continuous phase is more important for the introduction of the functionalities onto the external surface.

The paramount requirement for the additional functionalities is that they cannot interfere with the formation of the polymer and cannot react with the API or other payload components. We will take advantage of the functionalities located on the surface (“handles”) and react them to introduce moieties whose presence is necessary on the outer surface of produced capsules.

## Bifunctional Monomers Equipped With Additional Reactive Moieties

**Figure 2:**
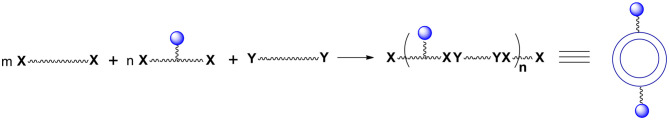
The number of “handles” (and therefore final surface moieties) on the capsules' surface can be controlled. To this end one must react bifunctional compound comprising Ys with a mixture of bifunctional compounds comprising Xs. This mixture contains compounds equipped and non-equipped with future handles (blue spheres).

Let us consider some selected examples of applicable monomers. Practically, they must be soluble either only in the aqueous phase or only in the oil phase. One possibility to accomplish the desired solubility is to utilize compounds (monomers) with reacting functionalities that are highly non-polar such as acyl or alkyl halides or highly polar such as carboxylates. Another option is to introduce ionic or non-ionic but highly hydrophilic units into the reacting molecules which will render them soluble in water. Alternatively, one can protect hydrophilic functional groups such as hydroxyl, amino, carboxylic acid, etc. with moieties that are highly hydrophobic such as those containing substituted aromatic or cyclohexyl rings. Importantly, of interest are bi(tri)functional, reactive compounds with one or more additional functionalities not involved in the interfacial process. However, bi(tri)functional compounds without these additional groups are also of interest since they may be used to control the number of external surface functional groups (Figure 2). To this purpose one can use a selected ratio of monomers containing “handles” (blue spheres) and monomers containing no “handles” during the shell formation. Varying the ratio of monomers equipped and non-equipped with additional moieties enables manufacturing capsules with a desired number of surface groups (Figure 2). The ability to control the density of handles on the surface is often crucial. It allows for surface moieties to be located sufficiently apart to minimize interactions between each other. Furthermore, it makes possible manufacturing capsules with a very small number of surface moieties (the average number can be as small as 1) which is necessary for some analytical applications.

There is a plethora bifunctional compounds applicable to IPCESCO. Particularly attractive are modified mono and oligosaccharides. It is because the carbohydrate literature describes a variety of differently protected polyfunctional compounds. It includes α,ω-bifunctional compounds with additional (protected) groups between α and ω positions. Here are a few examples of possible monomers and their synthesis (for brevity we did not include the chemistry of the protection of additional functional groups):

### Starting From 1,2:5,6-di-*O*-isopropylidene-α-D-glucofuranose 1 (Figure 3)

a) It is one of the most often utilized monosaccharide (glucose) derivatives. Let us introduce a protecting group into the position 3 (compound **2**). It can be highly hydrophobic if we need a water insoluble final product. Let us remove both acetonide groups and react the resulting polyhydroxy-compound with periodate. The formed dialdehyde **3** can be oxidized to diacid **4** (protected tartronic acid). The diacid salt can be reacted with appropriate dihalide or ditosylate to form the capsules' polyester shell. If a protecting group is highly hydrophobic, diacid (and its anion) will be practically insoluble in water. Of course, a protecting group can be removed or it can be hydrophilic. In such a case diacid (and its salt) will be water soluble.
b) Diacid **4** may be transformed into corresponding diacyl halide **5** which is a suitable monomer for the reaction with diols or diamines to form polyesters or polyamides.
c) Alternatively, dialdehyde **3** resulted from the periodate oxidation can be reduced to form diol **6**. The secondary OH group can be protected by either hydrophobic or hydrophilic group. Thus, we can have either water or oil-soluble triol with a protected hydroxyl group in the position 2. This approach is in many aspects better than the one starting from glycerol (via 1,3-benzylideneglycerol).
d) The OH groups in diol **6** can be transformed to ditosylate **7** or dihalide such as dibromide **8**. The product can be used as a reactant dissolved in the continuous or non-continuous phase to form the capsular shell composed of polyester and polyether.

**Figure 3:**
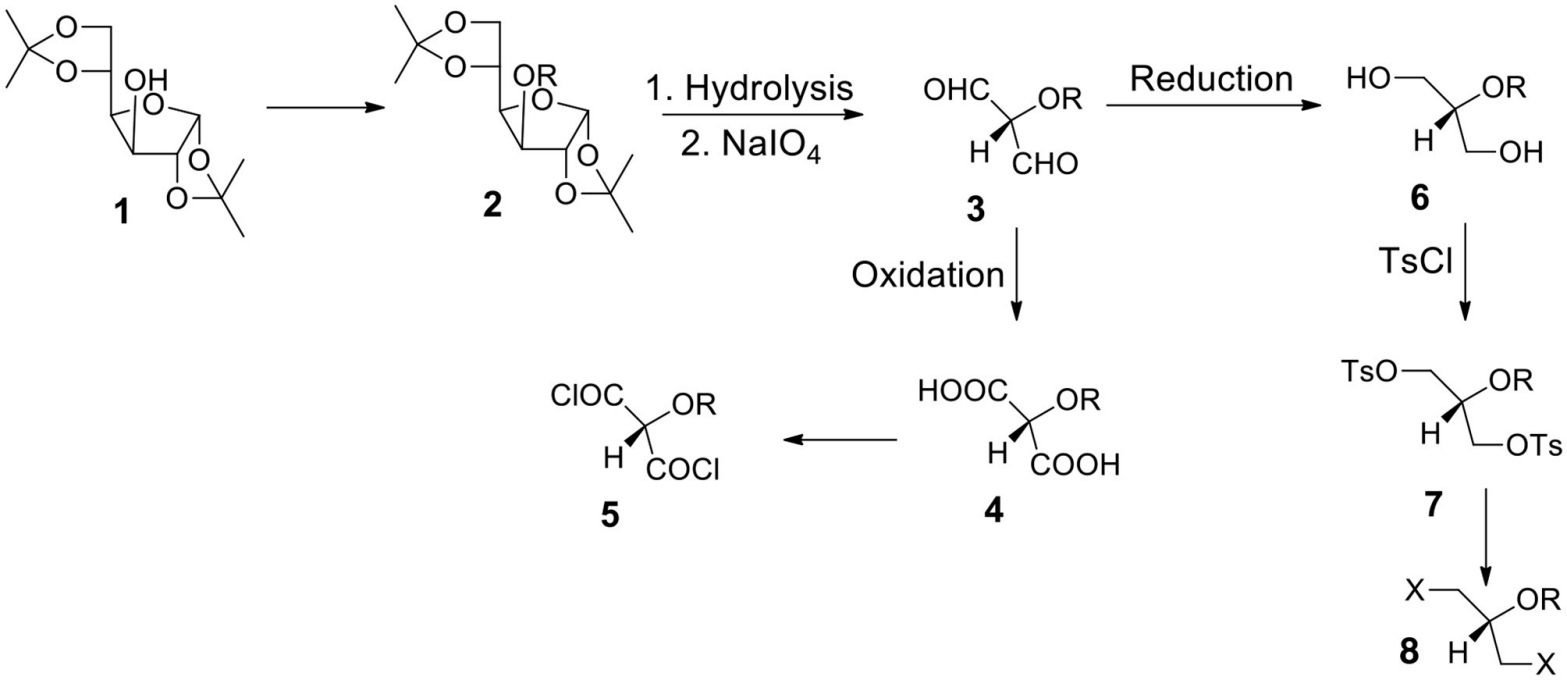
Synthesis of aqueous soluble and aqueous insoluble bifunctional compounds starting from 1,2:5,6-di-*O*-isopropylidene-α-**D**-glucofuranose **1**. Hydrolysis and periodate oxidation produce versatile (hydroxy-protected) dialdehyde **3** that can be transformed into hydrophilic or hydrophobic moieties equipped with various reactive groups.

### Starting From 3,4-*O*-isopropylidene-D-mannitol (Figure 4)

a) It is another easily available and broadly used monosaccharide derivative. It can be reacted with periodate. The resulting dialdehyde product **10** can be utilized as a monomer or can be reduced to diol **11** (1,2,3,4-butanetetraol with protected 2- and 3-OH groups). The same compound (with R configuration on both chiral carbon atoms) is also available from dimethyl L-tartrate **12**, non-expensive ester (MaGee et al., 2011). Also, both erythritol and threitol (non-protected tetraols) can be obtained by reducing corresponding aldotetroses. This diol can be reacted with bi(tri)functional acyl halide in the presence of a base such as pyridine or with bis alkyl halide in the presence of a strong base to form shell's polyester or polyether.
b) The oxidation of dialdehyde **10** produces 2,3-protected tartaric acid **13**. This acid (and its salt) is water soluble (unless ketone or aldehyde much more hydrophobic than acetone was used to form the starting material). Nota bene, isopropylidene and benzylidene derivatives of tartaric esters are commercially available. The reactant acid (sodium salt) can contain hydroxyl groups in a protected form. After the polymer (such as polyester) formation and deprotection we have hydroxyl groups on the surface. Formally speaking they derive from tartaric acid.

**Figure 4:**
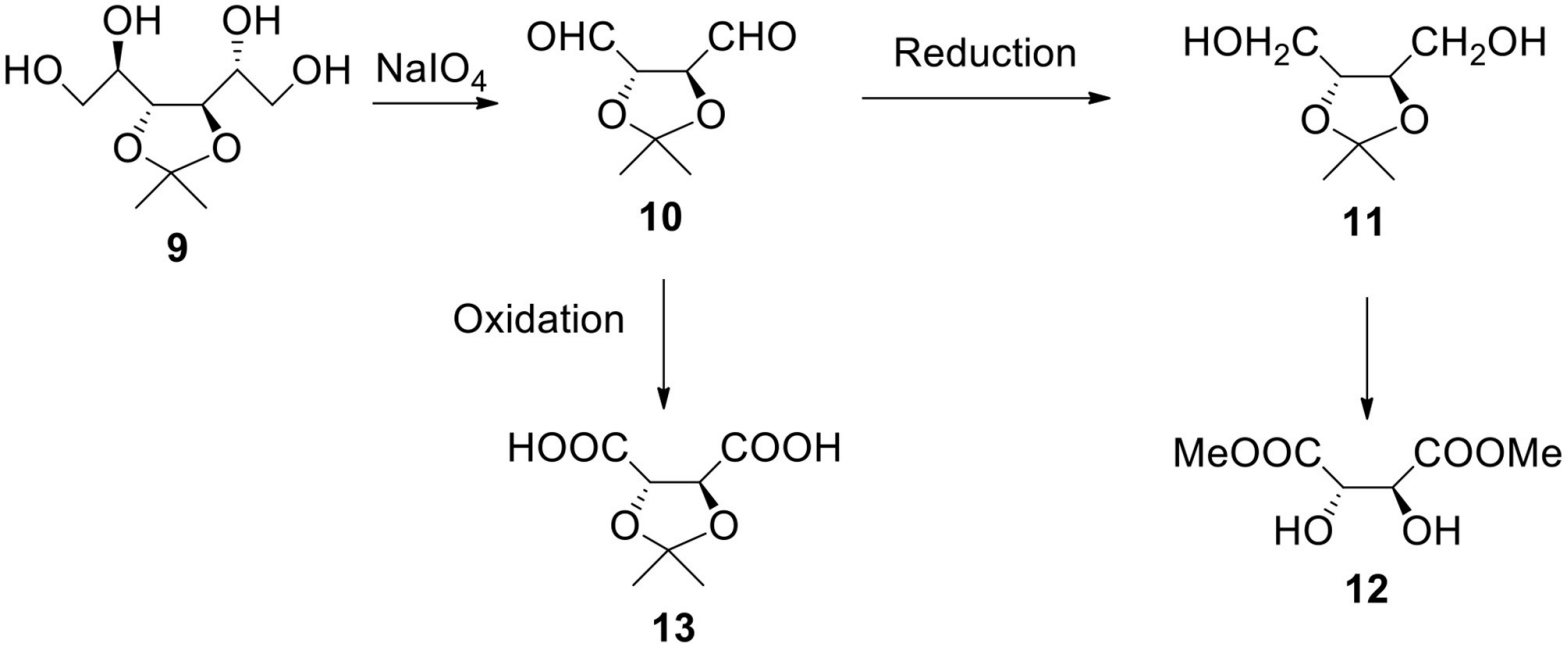
Synthesis of α,ω-bifunctional compounds (diols and diacids) starting from 3,4-*O*-isopropylidene-D-mannitol **9**.

### 5-Hydroxymethylfurfural-Based Bifunctional Compounds

**Figure 5:**
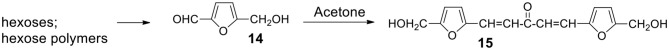
Useful α,ω-diol deriving from condensation of acetone with 5-hydroxymethyl furfural **14**.

5-hydroxymethylfurfural **14** is available from degradation of various mono and polysaccharides (Tong et al., 2010; van Putten et al., 2013; Alipour, 2016; Alipour et al., 2017). It can be transformed (via a Claisen-Schmidt condensation with acetone) into a very attractive bifunctional compound (diol) equipped with an additional α.β-unsaturated ketone **15** (Figure 5; West et al., 2008; Wu et al., 2016). Note that α,β-unsaturated ketone moiety can be an object of various Michael additions.

### Bifunctional Compounds Derived From Trehalose

**Figure 6:**
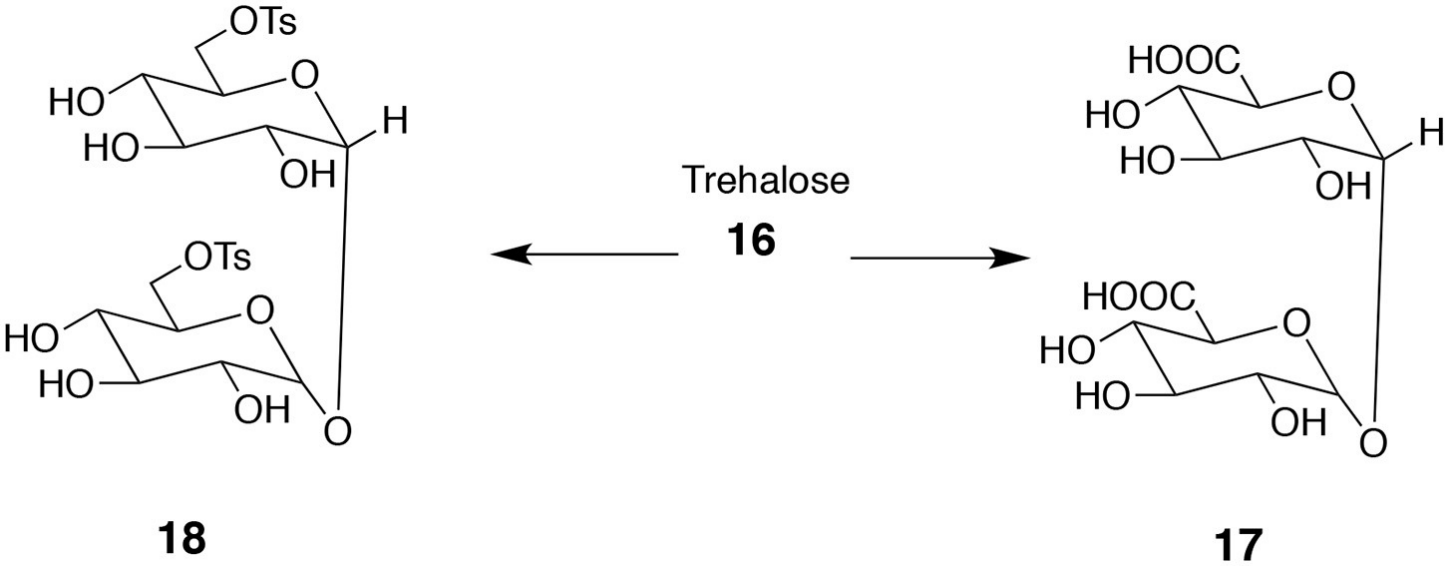
Attractive bifunctional compounds derived from trehalose. The difference in primary and secondary OH groups' reactivity allows for reacting only the former ones to form diacid **17** or ditosylate **18**.

Trehalose **16** (α-D-glucopyranosyl-α-D-glucopyranoside) is an excellent source of useful bifunctional monomers. It is a disaccharide consisting of two glucose units connected via C-1 at both monosaccharides. Note a high level of symmetry present in trehalose. One option is to transform primary hydroxyl groups into diacids **17** or ditosylates **18** (Figure 6; Birch and Richardson, 1968). The resulting product contains as many as six secondary hydroxyl groups that can be acylated or alkylated. Depending on the character of introduced substituents (hydrophilic or hydrophobic) the products can be either soluble or insoluble in water.

### α,ω-Bifunctional Compounds Derived From Glucoheptanoic Acid

**Figure 7:**
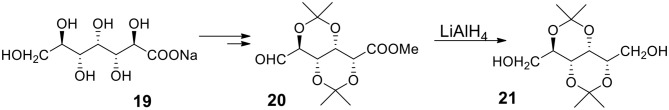
Attractive diol synthesized from glucoheptanoic acid.

Recently, Fleet, Yoshihara and coworkers (Liu et al., 2016) offered a very robust approach to some of the discussed compounds. The synthesis starts with easily available glucoheptanoic acid **19** (Figure 7) with all the OH groups protected as acetonides. The acid **19** can be transformed to a product containing aldehyde and ester functionalities in terminal positions (**20**). The LAH reduction provides diol with several protected hydroxyl groups **21**. The authors emphasize the great easiness of working with the described compounds since, due to the presence of isopropylidene moieties, the products are soluble in such hydrophobic solvents as hexane. The methodology allows to synthesize a variety of α,ω-bifunctional compounds containing several protected (as acetonides) or non-protected secondary hydroxyl groups from **20**. The synthesis of monomers for ICP or IAP is expected to be easier than synthesizing reported compounds because the desired bifunctional compounds must be highly symmetrical and carry same functionalities in α and ω positions.

### Sugar-Derived Dialdehyde

**Figure 8:**
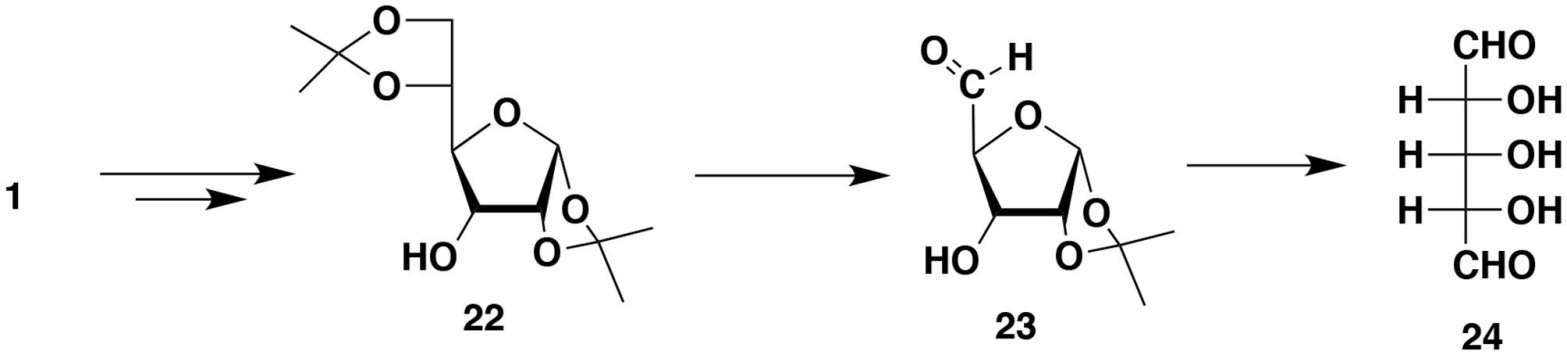
Miculka's synthesis of trihydroxydialdehyde **24**.

Miculka (1999) developed a very elegant synthesis of racemic ribose from 1,2:5,6-diisopropylidene-α-D-glucofuranose **1**. The synthesis consists of reversing the OH group configuration at C3 (diisopropylideneallofuranose **22**), the hydrolysis of one isopropylidene group followed by the cleavage at C5 (aldehyde **23**) and hydrolysis of the second isopropylidene group to form an interesting symmetric dialdehyde **24** with three hydroxyl groups. Borohydride reduction of one aldehyde group produces the desired (ribose) racemate. After protecting the OH groups dialdehyde **24** can act as a very effective IPCESCO monomer. However, note that the OH group protection is doable but by no means trivial since the dialdehyde exists as a mixture of furanose, pyranose and α and β hemiacetals (Figure 8).

### Tartaric Acid Derivatives

**Figure 9:**
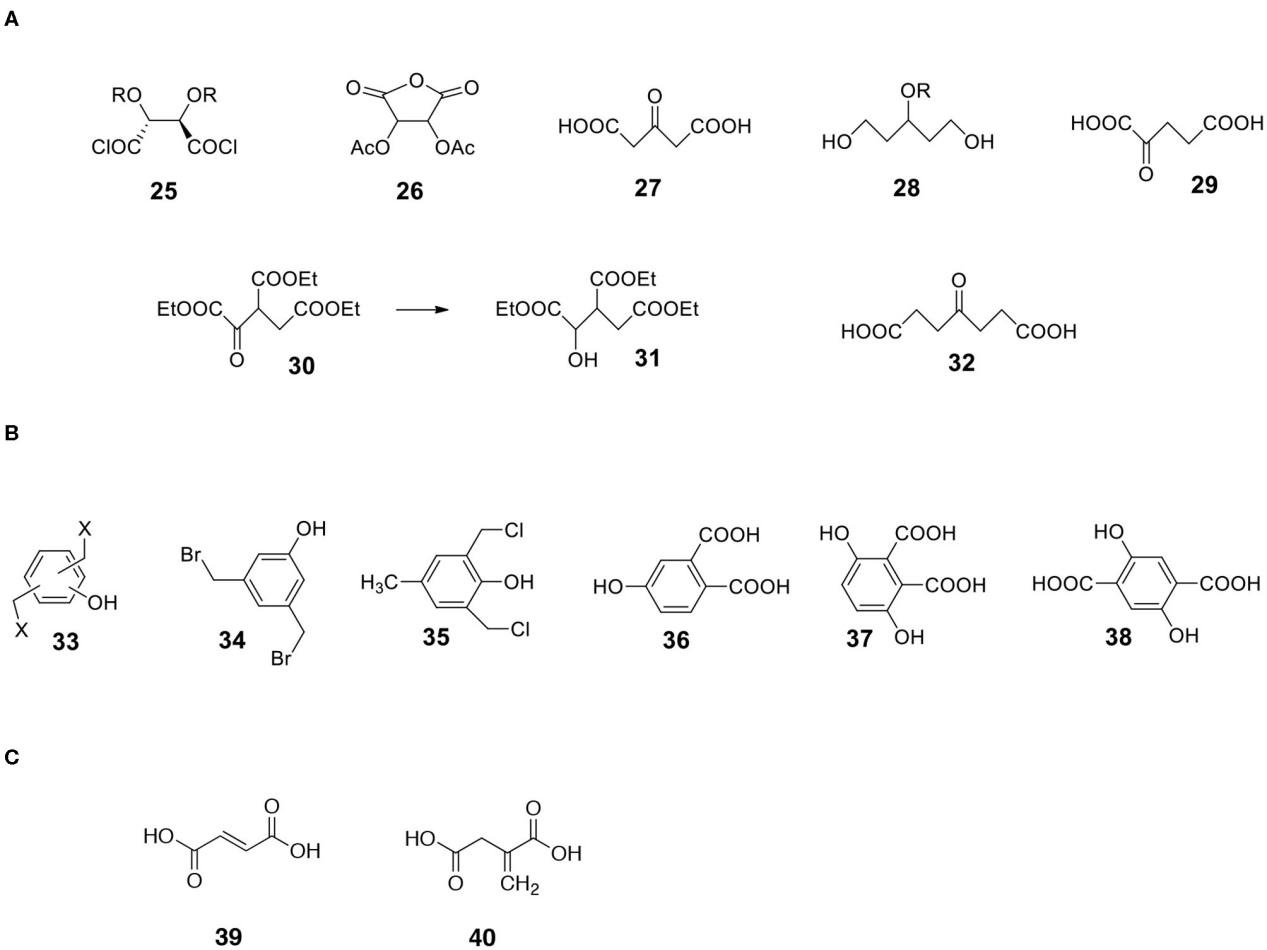
Structures of useful derivatives of aliphatic diacids and bifunctional aromatic compounds. **(A)** Aliphatic diacids. **(B)** Aromatic bifunctional compounds. **(C)** Unsaturated diacids.

Very useful bifunctional compounds can be synthesized not only from carbohydrates. Tartaric acid derivatives are interesting reactants. Hydroxy-protected tartaryl chloride **25** (Figure 9) is synthesizable (Choi et al., 1997; Ilmarinen et al., 2001). Other possibilities are diacyl tartaric anhydrides **26** (Bernaś et al., 2010) and already mentioned dimethyl tartrate **12** (MaGee et al., 2011).

### Bifunctional Aliphatic Acids With An Additional Carbonyl Group

a) 3-ketoglutaric acid **27** (acetonedicarboxylic acid) is available via decarbonylation of citric acid in neat sulfuric acid (Adams et al., 1925). The compound can be transformed into keto-protected or hydroxy-protected diacid and used as a monomer. The reduction of hydroxy-protected diacid offers interesting 1,3,5-triol **28** with a protected hydroxyl group in position 3 (Figure 9).
b) A similar compound, 2-ketoglutaric acid **29**, is also synthesizable (Bottorff and Moore, 1964). By the way, during the synthesis of 2-ketoglutaric acid, ester of triacid is an intermediate. It is triethyl oxalylsuccinate **30** (Figure 9). This ester can be easily reduced to form an example of triacid containing an additional OH group **31** (different from citric acid).
c) Another ketodiacid (4-oxoheptanedioic acid **32**) is commercially available (Figure 9).

### Aromatic Compounds {bis(halomethyl)benzenes and Aromatic Diacids}

Another category of non-carbohydrate-derived monomers includes bis(halomethyl)benzenes equipped with additional functionalities such as phenolic hydroxyl group **33** (Figure 9; Katritzky et al., 2006; Routasalo et al., 2008; Daumann et al., 2012). Such (water insoluble) compounds can react with salts of bifunctional carboxylic acids to form esters. Note that salts of carboxylic moieties can be formed without affecting the phenolic OH group. Thus, protecting the phenolic hydroxyl group may not be necessary in certain cases. Some bis(halomethyl)benzenes with phenolic OH such as 3,5-bis(bromomethyl)phenol **34** and 2,6-bis(chloromethyl)-4-methylphenol **35** are commercially available. Examples of useful substituted aromatic di-acids that are available include 4-hydroxyphthalic acid **36**, 3,6-dihydroxyphthalic **37** and 2,5-dihydroxyterephthalic acid **38** (Figure 9).

### Unsaturated Dicarboxylic Acids

Diacids containing a double bond are another category of useful monomers. There are a few common, natural products belonging to this group. They include maleic **39** and itaconic **40** acids (Figure 9). Note that the double bond is rather a non-reactive moiety and will likely survive most transformations leading to the formation of capsules. Finally, the double bonds present on the surface of capsules can be reacted (Michael addition) with appropriate thiols (Yamashita and Mukaiyama, 1985; Chanda and Ramakrishnan, 2015).

Note also, that it is sometimes advantageous to use compounds (monomers) containing more than two functional groups capable of entering polyaddition or polycondensation. Due to the resulting crosslinking the presence of a third or even fourth reacting functionality offers an improved strength of the polymeric shell. The kinetics of reactions of functional groups belonging to tri(tetra)functional compounds usually does not differ significantly from that of bifunctional compounds. Preferably, the tri or tetra-functional monomers are compounds with additional non-reactive groups (or moieties that can be protected such as **30** and **31**). Useful triacids include citric acid and oligo amino acids. There is a plethora of polyfunctional hydroxy compounds such as tri and tetrahydroxybenzenes and various mono and oligosaccharides. Some useful polyfunctional amines such as tris(2-aminoethyl)amine are commercially available. Obviously, oligopeptides are another category of applicable polyamino-compounds. Relevant trihalides can be produced from corresponding trihydroxy compounds.

There is one more point worth mentioning: very rarely the monomers should be enantiomerically pure. Thus, racemic or non-chiral monomers are usually acceptable, provided that they do not cause an adverse reaction in the body. When starting from chiral natural products we tend to produce pure stereoisomers. However, it is not important from the monomers standpoint. On the other hand, ligands attached to the external capsules' surface which are to be recognized by specific receptors must exhibit a specific stereochemistry fitting into the receptors' steric requirements.

## External Surface Manipulation

The interfacial polymerization process results in the formation of micro/nanocapsules containing (protected) functional groups (handles) we must transform into the required moieties. For simplicity we will discuss only those systems that took advantage of only one group not involved in the polymerization. There are several possible scenarios here. We will use a hydroxyl group as an example of a reactive chemical functionality (pink spheres in Figure 1):

### One Protective Group

**Figure 10:**
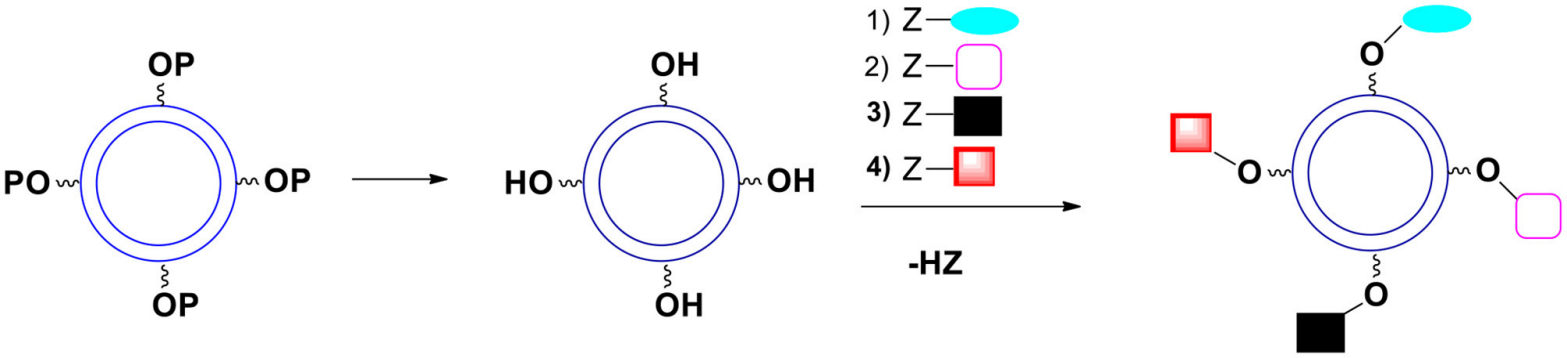
Deprotection of surface hydroxyl groups followed by a simultaneous introduction of surface moieties. P represents a protecting group. The kinetics of introduction of new surface groups should be more or less the same.

**Figure 11:**
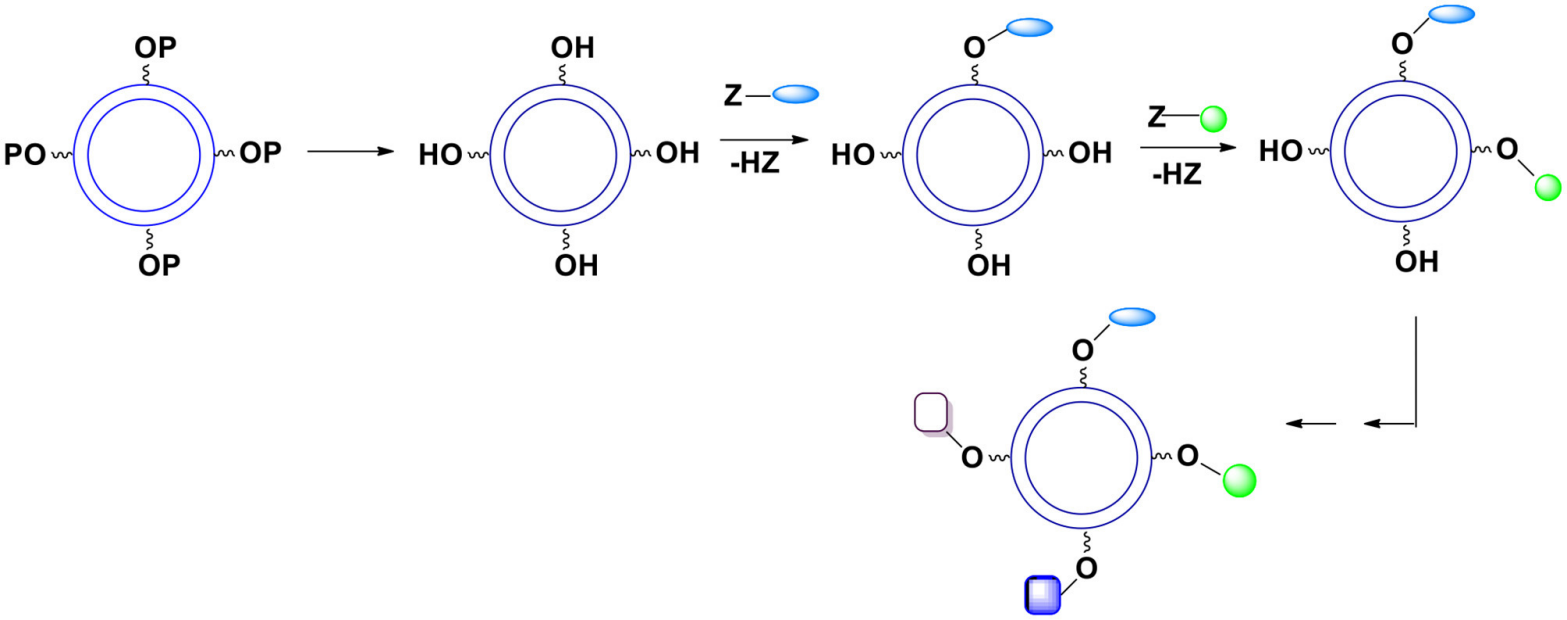
Deprotection of surface hydroxyl groups followed by a gradual introduction of surface moieties. P represents a protecting group. The relative kinetics of introduction of new surface groups is of no importance.

**Figure 12:**
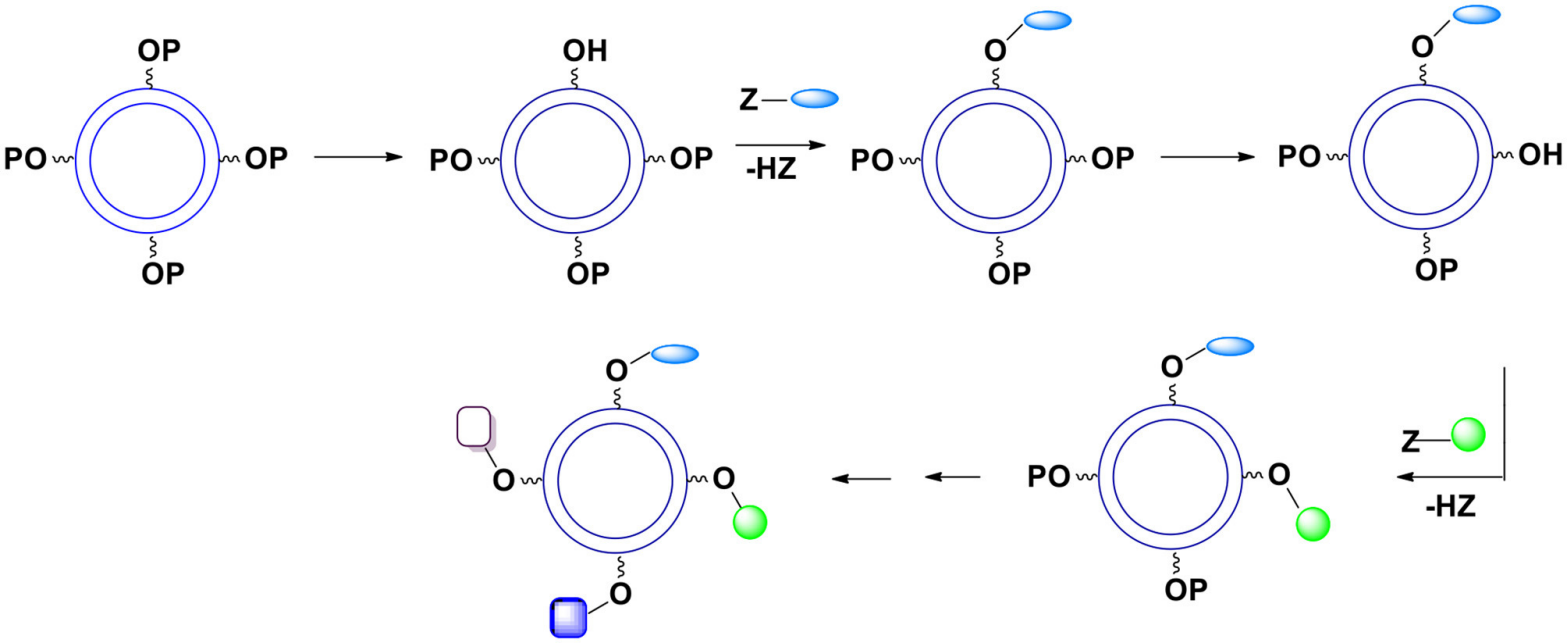
Gradual deprotection of surface hydroxyl groups followed by an introduction of surface moieties.

- All the OH groups are protected with the same protecting group during the shell formation. After the capsules are formed the protecting group is removed. If the moieties to be introduced onto the surface do not interfere with each other during the reaction, they can all be introduced in a one pot process (Figure 10). Since the kinetics of introducing various groups is often similar, one can control the ratio of desired groups on the surface.
- In a more typical case when introduced groups may interfere with each other during the reaction the surface moieties are introduced gradually (Figure 11).
- Theoretically, one can gradually remove a required number of protective units and replace them with the surface moieties (Figure 12) but this approach does not seem practical because the art of deprotection is less developed than the art of protection and at this point it is almost impossible to control the kinetics of deprotection.

### Several Protective Groups

**Figure 13:**
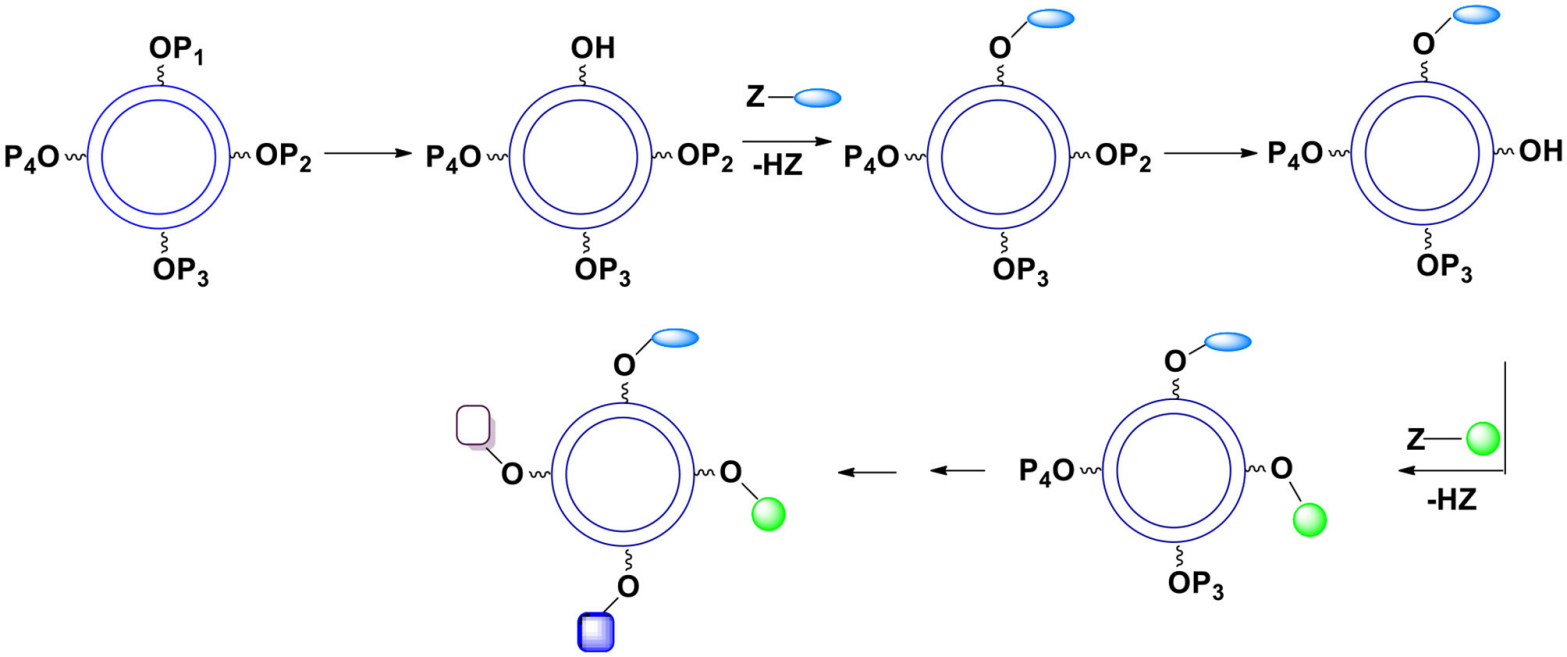
The use of various hydroxyl protective groups. Gradual deprotection of single protective groups followed by introduction of selected surface moieties.

A substantially better control of chemical functionalities on the external capsules surface can be accomplished when utilizing many different protecting groups. If their deprotection conditions differ, each protecting group can be exclusively removed when needed. In this case several monomers are employed during the formation of the capsules. They differ only in the units protecting the hydroxyl moiety. Since the presence of a protective group in the monomer practically does not affect the kinetics of the reaction of X with Y, the ratio of various protective groups on the surface can be well-determined. After the shell formation, OH groups are gradually deprotected and reacted with desired surface moieties (Figure 13). Hydroxyl groups can be protected with a variety of groups forming acetals, esters, ethers (including silyl or benzyl ethers), etc. All these groups require very different deprotection conditions leading to the same product (hydroxyl group). Furthermore, within a given category there usually are several subcategories. For example, various acetals and ketals require distinctive hydrolysis conditions allowing for the use of more than one acetal as an OH protective moiety that can be selectively hydrolyzed. A few years ago, we looked at impressive examples of selective deprotection of acetals and other protective groups (Bielski and Witczak, 2013).

### No Protective Groups

**Figure 14:**
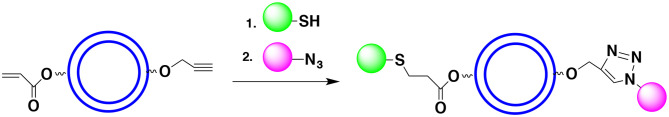
Use of click reactions to introduce surface moieties may allow avoiding the use of protective groups. In the example shown the terminal acetylene (via a reaction with propargyl bromide) and α,β-unsaturated ester (via a reaction with acryloyl chloride) are introduced. The Huisgen reaction and Michael addition put in place desired surface moieties.

**S**ometimes protecting the OH groups may not be necessary. It requires that either hydroxyl groups (or the functionalities hydroxyl groups have been transformed into) do not interfere with the shell formation and do not react with payload components. For example, one can react OH groups of the monomer with propargyl bromide to form propargyl ether. The propargyl group is not considered a protecting group (it cannot be easily deprotected). However, it can form a cyclic triazole (click product) when reacted with the azide group. Similarly, the monomer's OH group may be reacted with acryloyl halide to form an α,β-unsaturated ester (non-reactive under many coupling reaction conditions). This functionality can potentially enter the Michael addition with thiol to form a thioether (click product). Thus, the hydroxyl groups (non-involved in the polymerization) of the monomer can be reacted with rather exotic, non-reactive species that will not interfere with the polymerization process but will be ready for the surface functionalization (Figure 14). Since the reactants of both click reactions do not interfere with each other we can introduce all the reactants simultaneously and form a surface with two or more functionalities in a one pot process. Note that introduction of propargyl and acryloyl moieties can take place after the shell formation provided that hydroxyl groups do not affect the polymerization and do not react with the capsules core components.

We can conclude that manufacturing reservoir type micro/nanocapsules of well-controlled size, equipped with a predetermined number of functional groups on the surface is doable. These functional groups will act as “handles” to be coupled with selected chemical moieties. In turn, these moieties will ensure the necessary behavior of capsules during transport of the active to the desired destination or will allow for coupling specific antibodies to the capsule's surface for the detection, quantification and potential removal of various pathogens. Note that the discussed methodology offers an important benefit: while (protected) “handles” are present everywhere in the shell, only handles on the external capsules surface are available for the introduction of selected moieties. Since potential surface units such as antibodies or moieties recognized by specific receptors may be highly valuable, it is important to be able to use minimal quantities of these units.

Interfacial polymerization is not the only methodology enabling introduction of functionalities onto the capsules surface. Grafting is another possibility (Zheng et al., 2012; Frere et al., 2013). However, interfacial polymerization offers much better control of the surface architecture.

## Polymeric Shell Forming Reactions

At this point it seems appropriate to consider what chemical moieties can act as X and Y. The following list is by no means exhaustive. It includes reactions that have been and can be taken advantage of during the interfacial condensation or addition polymerization.

### A. POLYCONDENSATIONS

1. Acyl halide + primary or secondary amine → polyamides
  **RCOCl** **+** **R'NH**_**2**_ **→** **RCONHR'** **+** **HCl**
2. Acyl halide + alcohol (including phenol) → polyesters
  **RCOCl** **+** **R'OH (including ArOH)** **→** **RCOOR'** **+** **HCl**
3. Sulfonyl (or phosphonyl) halide + primary or secondary amine → polysulfonyl (or polyphosphonate) amides
  **RSO**_**2**_**Cl** **+** **R'NH**_**2**_ **→** **RSO**_**2**_**NHR'** **+** **HCl**
4. Sulfonyl (or phosphonyl) halide + alcohol (including phenol) → polysulfonyl (or polyphosphonate) esters
  **RSO**_**2**_**Cl** **+** **R'OH (including ArOH)** **→** **RSO**_**2**_**OR'** **+** **HCl**
5. Alkyl halide (or tosylate) + alcohol in the presence of a base → polyethers
  **RX (or ROTs)** **+** **R'OH** **→** **ROR'** **+** **HX**
6. Alkyl halide + amine to → poly(alkylated amine)
  **RX** **+** **R'NH**_**2**_ **→** **RR'NH** **+** **HX**
7. Alkyl halide (or tosylate) + carboxylate → polyesters
  **RX** **+** **R'COO**^**−**^ **→** **R'COOR** **+** **X**^**−**^

### B. POLYADDITIONS

1. Isocyanate + alcohol (thiol) → polyurethanes
  **RNCO** **+** **R'OH** **→** **RNHCOOR'**
2. Isocyanate primary (secondary) amine → polyureas
  **RNCO** **+** **R'NH**_**2**_ **→** **RNHCONHR'**
3. Isothiocyanate + alcohol → polythiouretanes
  **RNCS** **+** **R'OH** **→** **RNHCSOR'**
4. Isothiocyanate + primary (secondary) amine → polythioureas
  **RNCS** **+** **R'NH**_**2**_ **→** **RNHCSNHR'**
5. Thiol + α,β-unsaturated carbonyl compound → polythioethers
  **RSH** **+** **CH_2_ = CH-COR'** **→** **RSCH**_**2**_**CH**_**2**_**COR'**
6. Azide + terminal acetylene → poly(cyclic triazoles)
  **RN**_**3**_ **+1-alkyne** **→** **cyclic triazole**
7. Thiol + unsaturated compound to → polythioethers
  **RSH** **+** **CH_2_ = CHR'** **→** **RSCH**_**2**_**CH**_**2**_**R'**

The above list shows some of possible reactive functionalities in bifunctional (trifunctional) monomers. All these functionalities can be synthesized starting from units present in carbohydrates. A plurality of specific functionalities applicable to the discussed polymerization has been used to form various bioconjugates. Of course, the specific processes are useful only if appropriate derivatives of bifunctional and trifunctional monomers are available or can be easily synthesized (Hermanson, 2013). It is worth noting that some of the listed polyadditions {isocyanate + thiol (Hensarling et al., 2011), thiol + α,β-unsaturated carbonyl compound (Nair et al., 2014), azide + terminal acetylene (Lutz and Zarafshani, 2008; Tron et al., 2008), thiol + non-conjugated unsaturated compound (Dondoni and Marra, 2012; Lowe, 2014) belong to the click reactions.

As it was already emphasized the monomers must be soluble either in oil or in water. It requires that not only reactive functionalities and main chains but also the future handles have to exhibit the appropriate (hydrophobic or hydrophilic) character. This asks for a skillful manipulation of protective groups, particularly when modified mono or oligosaccharides are used to synthesize monomers for the discussed processes. It is because carbohydrates often contain a plurality of hydroxyl groups. The presence of several non-protected hydroxyl groups ensures the compound solubility in the aqueous phase. Alternatively, if these hydroxyl groups are protected as highly hydrophobic ethers, esters or acetals, the resulting modified carbohydrates are oil-soluble. Thus, water soluble and insoluble say dibromides or ditosylates and water soluble and insoluble salts of dicarboxylic acids can be synthesized starting from mono or disaccharides.

The choice of monomers for the polymerization process must depend on specific circumstances. Usually, the most important are the chemical and physical properties of the payload components.

## Manipulation of Capsules' Surface

Choosing the strategy leading to the formation of capsules with necessary moieties on the surface is not trivial. Usually, groups that are to be present on the surface of capsules should not be present in monomers forming the capsules' shell.

It may be important to know how many moieties of different types there are on a surface of an average capsule. Usually, calculations based on the expected yields are sufficient. If more reliable data are necessary one can react “handles” with reactants that introduce measurable properties onto the surface such as UV activity, radioactivity, fluorescence, etc.

It is worth adding that early in this century professor Carolyn Bertozzi introduced an exceptionally fruitful concept of bioorthogonal chemistry into the area of chemistry applied to living organisms. One of the objects of interest of bioorthogonal chemistry is decorating the surface of cells with various moieties. Many of the concepts and protocols developed as a result of the relevant research can be directly applied to manipulating of the capsules' surface (Sletten and Bertozzi, 2009; Kennedy et al., 2013; Hudak and Bertozzi, 2014).

Another aspect of the surface design that should be considered when planning the surface of capsules is a possible interaction(s) between surface moieties. They must be as minimal as possible. It can be accomplished by:

- Controlling the number of “handles” on the surface. Decreasing the number of surface “handles” decreases the number of functional groups on the surface and consequently increases the distance between the surface moieties.
- Controlling the distance of functional groups from the surface. It may require that spacers of varying length are employed.
- Protecting the functional groups that can potentially interfere with each other. The special protecting groups must be such that they will get deprotected at the desired destination. Note that the protecting groups discussed here differ from those discussed earlier in that they must survive in the medium such as the aqueous matrix or blood and not polymerization.

Spacers represent another tool worth mentioning. There are at least four reasons to introduce spacers (linkers) onto the external surface of the capsules:

a/ to minimize various non-specific binding of capsules,
b/ to ensure that the transport facilitating moieties are sufficiently away from the capsules' surface.
c/ to affect hydrophilicity or hydrophobicity of capsules surfaces.
d/ to minimize interactions between moieties present on the same capsule by increasing the distance between these moieties.

The spacers may be either hydrophilic or hydrophobic. The hydrophobic ones can be made both of aliphatic and aromatic units (Licea-Claveríe et al., 2007). The most common hydrophilic spacers are composed of ethylene glycol units, however ethyleneamine or monosaccharide units may be equally economic (Takahashi et al., 2006; Jiao et al., 2007; Shibata et al., 2010). Recently, Kokkoli et al. showed that the choice of the spacer used to link the hydrophobic and hydrophilic blocks of an aptamer-amphiphile can dramatically influence their self-assembly (Pearce et al., 2014). It is evident that the type and size of spacers should be optimized for specific applications.

## Applications

Micro/nanocapsules manufactured using IPCESCO should find a variety of applications. Here are a few possibilities:

**A. Drug Delivery**
  An effective delivery of active pharmaceutical ingredients (APIs) to diseased organs or tissues is crucial for the success of the employed therapy. However, it seems that our efforts are not sufficiently focused on ensuring that drug molecules reach the target. A recent paper by Canadian researchers (Wilhelm et al., 2016) can serve as an excellent illustration of this fact. It turns out that only a small number of medicinally active (against various cancers) molecules delivered in nanoparticles reach the desired destination. Even if this number is higher for other applications, there is no doubt that we must dramatically decrease the losses of the API during drug molecules' delivery. We hope that employing IPCESCO capsules will help addressing some of the relevant issues by enforcing safe transport of capsules in the blood to desired destinations followed by the payload release at this location. The presence of selected moieties on the external capsule surface should prevent the formation of agglomerates, minimize capsules degradation, facilitate crossing various barriers including blood-brain barrier, allow for “parking” of capsules in desired locations, etc. The benefits of equipping the surface of micro(nano)capsules with several functionalities for controlled delivery of drugs include:

- The solubility of a drug in aqueous systems is a very important factor determining its evaluation. However, the solubility of a drug to be encapsulated does not matter–at least during transport. Thus, insoluble drugs can be encapsulated, and with the appropriately functionalized (hydrophilic) surface can be transported throughout blood or other body fluids.
- Introducing desired properties to the capsules, such as the ability of being recognized by specific receptors, does not have to affect the structure of the drug. The necessary chemical functionalities can be present on the capsules' surface.
- When a biologically active compound is conjugated directly to a selected moiety (pro-drug), the molar ratio of the drug to the moiety is almost always 1:1 (sometimes it can be 2:1). However, when an active compound is encapsulated, the number of moieties present on the surface of a single capsule is significantly larger than 1, but still much smaller than the number of molecules of the active inside the capsule. Consequently, a small number of receptors can attract a small number of capsules but a very large number of molecules of the encapsulated drug.
- Since the design and chemical structure of the capsules' surface can be such that it is the most convenient for the given application, it is almost always easier to couple the required moieties to the capsules' surface than directly to the molecules of the active compound.
- It is exceptionally rare that a molecule of the active compound (API) has many chemical “handles” one can take advantage of and couple several functionalities to the molecules of the active. Since capsules are much larger than molecules of active chemicals it is possible to have many “handles” on the surface of the micro(nano)capsules, and thus, attach several different functional groups facilitating the capsules' transport.
- The preferred method of manufacturing capsules with a multifunctional surface, IPCESCO, allows for a good control of the shell thickness. This enables controlling the rate of the release of the active compound after the capsules reached the target destination. When such control of the rate of the release of the active pharmaceutical ingredient (API) is necessary the release stimulus of choice is the use of enzymes. If the release takes place at the location and the rate of the release is similar to the rate of the active reaction with the specific receptors, then most of the active is not degraded but utilized to do the required job.
- When the payload release takes place only at a targeted location and the release kinetics is controlled, much less of the active compound is needed. This significantly improves the economics of the drug's application while minimizing side effects for the patient.

There are several factors determining the design of the surface of capsules for drug delivery. Probably the most important factor is the choice of the way the drug is administered. The intravenous administration allows for larger capsules (microcapsules or nanocapsules) and in general the surface requirements are less stringent than those for oral administration. The oral administration is always more convenient from the patient standpoint. However, it allows only for nanocapsules and even those must be relatively small.

Another factor to be considered is the release mechanism. The degradation of the shell by enzymes is not always possible. Since IPCESCO produces only reservoir capsules it seems that the most beneficial release of the payload is a rapid rupture of the capsules shell at the desired location. There are a few ways of accomplishing it. For example, the rupture can be triggered remotely using selected physical methods such as focused ultra-sound, electric or magnetic field, etc.

The shell of reservoir capsules must not be destroyed before the capsules reach the desired destination. There are two ways of ensuring safety of the shell during the transport. One option is that capsules' shell is practically indestructible (or the degradation is rather slow) in blood but can be degraded using very selective methods such as specific enzymes located only at the desired destination. In this case, relatively large capsules can be utilized. The specific enzymes may be introduced directly into the target location (perhaps in the encapsulated form) or they can be there naturally.

An alternative approach takes advantage of reservoir capsules with a shell that practically cannot be degraded by chemicals present in environments the capsules will be in. It does not mean that the shell cannot be ruptured using some mechanical means such as ultra sound. Thus, after capsules reach the desired destination, one of possible physical methods is remotely applied causing the shell's rupture. Consequently, a payload is released. Physical methods other than ultra sound such as electric and magnetic fields can be utilized as well but that requires a presence of special components in the payload. After the release of an API there is a leftover polymer (shell) which is not degradable. Thus, the question concerning the excretion of the shell material from the organism needs answering. One possible response is that the ruptured shell must be sufficiently small to be below the renal exclusion limit (REL) (as far as we are aware at the moment it is considered to be about 6 nm) (Longmire et al., 2008). Note that the size of the ruptured shell is significantly smaller than the size (diameter) of the original nanocapsules. The REL value will have to be determined for specific materials and applications. However, it is worth noting that for molecules of API exhibiting a molecular mass of say 200 Daltons, a small nanocapsule with a diameter of only 20 nanometers can still carry a payload of several thousand molecules ({20 nm}^3^ = 8000 nm^3^; the size of a single molecule of glucose (Mw = 180 D) in the cyclic form is about 0.85 nm in each direction. The number of molecules in nanocapsules will be little smaller for a typical low molecular API of MW = 500 D.

What are degradable functional groups that can be formed when reacting the monomers? Unfortunately, the picture is not very clear. For obvious reasons researchers utilize almost always biodegradable polymers and those are found in the literature. The data on non-degradable polymers are much more scarcely available. Very generally we can say that the following chemical linkages are biodegradable: amide, ester, carbonate, anhydride, urethane, orthoester (Coelho et al., 2010). The non-biodegradable category includes acrylate polymers, polyurethanes, silicone rubber, poly (ethylene vinyl acetate) (Coelho et al., 2010; Fu and Kao, 2010).

The crucial question determining the strategy for capsules' formation is—what moieties should be present on the surface. For oral delivery functionalities (absorption enhancers) that facilitate transfer of nanoparticles from intestine to plasma are one such category (Lundquist and Artursson, 2016; Zhu et al., 2016). Other group addresses challenges created by various barriers (Date et al., 2016) such as blood-brain barrier preventing the entrance of foreign bodies into specific organs.

One of the most important surface moieties are those that are recognized by specific receptors at the targeted location. While large molecules are also of interest it seems that particularly attractive ligands belong to small molecules. In a relatively recent, excellent review Nicolas and coworkers offer an exhaustive table of receptors and corresponding ligands that have been used in drug delivery applications (Nicolas et al., 2013). The table includes several small molecules ligands such as folic acid, biotin, curcumin, selectin, alendronate, glutathione and mono and disaccharides such as galactose, glucose, mannose, sialic acid, galactosamine, and lactose. Recently, it has been shown that dual ligand targeting may be highly effective (Alexander-Bryant et al., 2013). It strengthens the importance of our ability to introduce many moieties onto the capsules' surface. Another aspect that must be considered when designing the surface of the capsules is their hydrophilicity and hydrophobicity (Alexander-Bryant et al., 2013).

B. **Ultra Sensitive Detection and Quantification**
  Another area of useful applications of capsules with controlled surface is in ultrasensitive detection. Consider micro/nanocapsules equipped with antibodies for specific pathogens on the surface and selected reporter molecules (RMs) in a payload. The number of these antibodies can be controlled. We want this number to be such that a capsule forms on the average a conjugate with one pathogen. The number differs for various circumstances and employing the proper number of antibodies may require some brief experimentation. After the matrix of interest (say a sample of contaminated water) was exposed to an excess of capsules, the capsules which formed conjugates with pathogens must be separated from those which did not. In this context the ratio of a mass of a capsule coupled to a pathogen to a mass of an unreacted capsule becomes important (m_MC_ = mas of microcapsule; m_p_ = mass of pathogen)

R = (m_MC_ + m_P_)/m_MC_

If R differs significantly from 1.0 we can take advantage of centrifugation as a separation method. The minimal value of R should be not less than <1.3 or so. Note that a mass distribution of micro/nanocapsules is not perfect. The difference between a mass of a capsule coupled to a pathogen and a mass of an unreacted capsule depends on a few factors but the most important is the size of the pathogens. Such notorious pathogens as viruses are considered to be between 20 and 400 nm in diameter. Bacteria are 400 nm or larger. Manipulating the size of micro/nanocapsules allows for the difference in the two masses to be such that R is 1.3 or more. Thus, if we are to measure very small viruses (20 nm) we can hardly employ nanocapsules with a diameter of 30 nm (if density of capsules and viruses is about the same R = {(30 nm}^3^d/(30 nm)^3^d + 20 nm}^3^d/{30 nm}^3^d = 1.3). In most cases 30 nm capsules carry an insufficient number of reporter molecules and centrifugation is not the suitable separation method. However, for large pathogens (say 700 nm in diameter) separating coupled and empty capsules should be possible even for capsules as big as 1000 nm (R = {1000 nm}^3^d/ + 700 nm}^3^d/{1000 nm}^3^d = 1.3. 1 micrometer (1000 nm) capsules can carry millions of RMs. In this case centrifugation may be a method of choice when separating coupled and non-coupled capsules. Additionally, one must consider the size/mass distribution of capsules. A very similar approach can be taken advantage of if we can introduce one nanomagnet into the payload of each capsule and couple a nanocapsule to each pathogen. The size distribution of such magnets can be good (10%). Of course, the separation of reacted and non-reacted capsules requires the use of a magnetic field.

**Figure 15:**
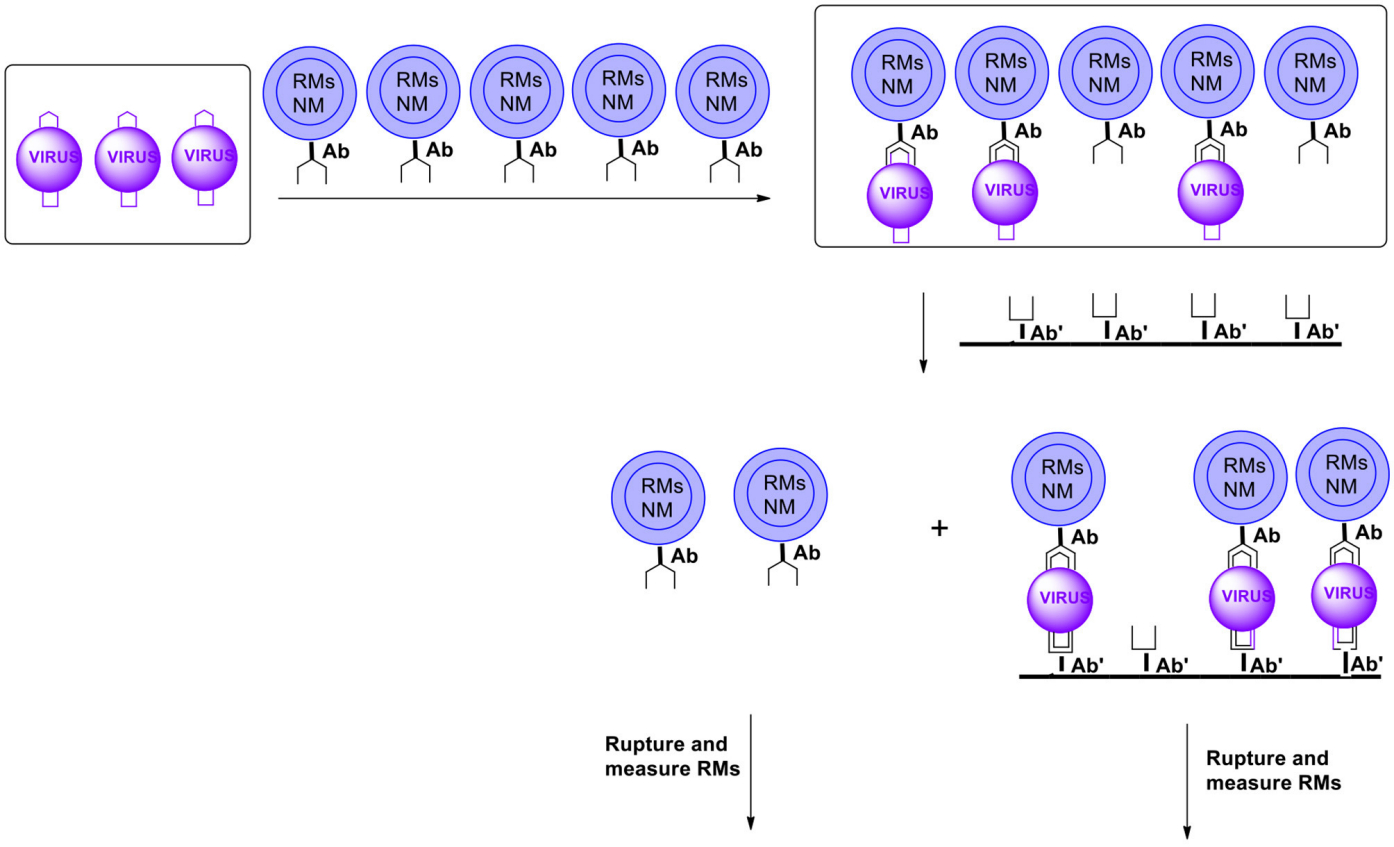
Schematic presentation of possible ultra-sensitive detection and/or quantitation using microcapsules (RMs, reporter molecules; NM, nanomagnet; Ab, antibody). For simplicity microcapsules contain antibodies for only one pathogen (virus). Microcapsules are additionally equipped with moieties such as those carrying hydrophilicity, preventing agglomeration and non-specific binding, etc. (not shown).

Another methodology enabling separating reacted from non-reacted capsules is the membrane filtration. In this case, capsules are equipped (on the surface) with antibodies recognizing the analyte (pathogen) of interest and other moieties such as those facilitating movement in the matrix (usually water), inhibiting the formation of agglomerates or minimizing the so called non-specific bonding. After the formation of conjugates antibody-antigen the capsules are introduced into the membrane containing on the surface antibodies for the same pathogens but utilizing another part (epitope) of the pathogen for coupling. Capsules with coupled pathogens get conjugated to the membrane surface via a (free) unit of a pathogen. Those capsules that do not contain pathogens will get through (Figure 15). Note that only pathogens coupled to both antibodies will stay on the membrane. It will minimize the so called non-specific binding.

After the separation both categories of capsules are ruptured to release their payload of reporter molecules. Measuring both sets allows for internal control of the results. Examples of reporter molecules include any easy detectable compounds such as UV or visible light dyes, fluorescent, luminescent, phosphorescent compounds, quantum dots, etc. It is worth adding that for reporter molecules with a typical molecular mass of say 250 Daltons a single microcapsule with a diameter of 1 micron can have a payload of more than 10^8^ RMs. If on the average one capsule is coupled to one antigen, the presence of one unit of the analyte is equivalent to releasing 100 million reporter molecules. Thus, the amplification is in the order of 10^8^.

C. **IPCESCO nanocapsules with nanomagnets**
  Micro/nanocapsules whose payload includes ferromagnetic units can be a/ directed inside the matrix, and b/ easily separated from all other particles not exhibiting affinity to magnetic fields. These properties can be taken advantage of in removing pathogenic bacteria or other microorganisms from most water-based matrices. For this purpose, microcapsules containing selected antibodies and other moieties on the surface are introduced into the pool. Additionally, the capsules have nano-magnets inside the payload (or attached to the capsules surface). Introducing nano-magnets into the payload or onto the surface of capsules is rather trivial when using IPCESCO. After the selected time period during which the formation of conjugates between capsules and pathogens had been completed, the water is pumped through the enclosure to which a powerful magnet had been attached. The capsules together with selected pathogens are removed from the system.

Possibly, a similar approach can be utilized during dialysis. It must be emphasized that ferromagnetism is a micromolar phenomenon. Nevertheless, ferromagnetic units as small as a few tens of nanometers are available and commonly used.

## Additional Comments

The obvious requirement for brevity is a good excuse for not addressing certain important factors that should be considered when designing nanocapsules for a specific application. Thus, we want to mention only some additional issues worth looking into. While controlling the location and type of individual chemical moieties on the surface of micro/nanocapsules is of major importance, one should not disregard capsules' surface general character. It still can be hydrophilic or hydrophobic. Both options may be necessary for specific circumstances. Besides employing covalently attached hydrophilic/hydrophobic units, one can also take advantage of non-covalent interactions. Similar considerations can be applied when a specific hydrophilic/hydrophobic factor is required or when the capsules' surface should carry a charge. Also, the presence of specific moieties on the capsules' surface does not preclude the use of stabilizing molecules such as PEGs, DNA or albumin to prevent aggregation or agglomeration of particles (Oh and Park, 2014). Furthermore, recent studies show rather surprising results with regards to the importance of such nanoparticles' properties as size and shape (Oh and Park, 2014; Shang et al., 2014).

Recently, a concept of “green nanomedicine” has been introduced (Jahangirian et al., 2017). As authors say “most of the developments with green processes have led to materials of low toxicity and high biocompatibility. As such, green chemistry methods have helped to avoid one of the biggest complications faced with today's NP (nanoparticles) drug delivery systems: toxicity.” We believe that when practicing the discussed methodology of manufacturing nanocapsules we should use principles of green chemistry whenever possible. In most cases it will translate to practicing “green nanomedicine.”

The knowledge of the patient's genotype has become more and more important when choosing the most effective API. It may take a few years but we expect that soon physicians will want to know the patient's genotype before selecting the optimal capsule's type for a given drug. We do not expect the capsular surface to be personalized i.e., that the surface will be adjusted to a specific patient. It is much more likely that a given drug will exist in 3 or 6 different versions. Each version will contain the same payload but will have a specific design of the surface.

## Conclusions

IPCESCO is a robust method of micro/nanocapsules' manufacture which allows for a very effective control of the capsules' external surface. In particular, it makes possible an introduction of a well-defined number of selected moieties. These moieties may prevent degradation of API during its transport or “parking” within the body, prevent formation of agglomerates, facilitate crossing various barriers, etc. Similar capsules may deliver nutrients to plants. Another type of micro/nano capsules carrying reporter molecules may be equipped with specific antibodies on the surface allowing for ultra-sensitive detection and quantitation of various pathogens. Adding nano-magnets into the discussed capsules payload may enable removal of pathogens from matrices.

## Data Availability Statement

The original contributions presented in the study are included in the article/supplementary material, further inquiries can be directed to the corresponding author/s.

## Author Contributions

All authors listed have made a substantial, direct and intellectual contribution to the work, and approved it for publication.

## Conflict of Interest

JN was a consultant for Chemventive LLC. The remaining authors declare that the research was conducted in the absence of any commercial or financial relationships that could be construed as a potential conflict of interest.
